# The significance of cone beam computed tomography for the visualization of anatomical variations and lesions in the maxillary sinus for patients hoping to have dental implant-supported maxillary restorations in a private dental office in Japan

**DOI:** 10.1186/1746-160X-10-20

**Published:** 2014-05-28

**Authors:** Kazunobu Shiki, Tatsurou Tanaka, Shinji Kito, Nao Wakasugi-Sato, Shinobu Matsumoto-Takeda, Masafumi Oda, Shun Nishimura, Yasuhiro Morimoto

**Affiliations:** 1 Yuugao Dental Office, Tokyo, Japan; 2 Division of Oral and Maxillofacial Radiology, Kyushu Dental University, 2-6-1 Manazuru, Kokurakita-ku, Kitakyushu 803-8580, Japan; 3 Center for Oral Biological Research, Kyushu Dental University, Kitakyushu, Japan

**Keywords:** Cone beam computed tomography, Maxillary sinus, Dental implants, Panoramic tomograph

## Abstract

**Objectives:**

The purpose of the present study was to elucidate the significance of cone bean computed tomography (CBCT) for patients hoping to undergo implant-supported restorations of the maxilla. Therefore, two studies were planned. One was to compare the prevalence of anatomic variations and lesions in the maxillary sinus on CBCT of patients hoping to undergo implant-supported restorations of the maxilla with that in patients with other chief complaints in a private dental office in Japan. The other study was to elucidate the limitations of panoramic radiographs in the detection of anatomic variations and lesions in the maxillary sinus.

**Study design:**

Sixty-one pairs of panoramic radiographs and CBCT were retrospectively analyzed in two groups of patients, those who hoped to undergo implant-supported restorations in the maxilla (Implant group) and those who did not (Non-implant group). The presence of anatomic variations and lesions in the maxillary sinus were analyzed.

**Results:**

The detection rate of mucosal thickening was significantly higher in the Implant group than in the Non-implant group. The detection rates for the features analyzed were significantly lower on panoramic radiographs. In particular, the detection rates of internal and anterior locations of some features were noticeably lower on panoramic radiographs. A significant relationship was found between the change in the detection rate on panoramic radiographs and the widths of mucosal thickening or the lengths of the major axis of SOLs in the maxillary sinus. If the width of mucosal thickening or the length of the major axis of SOLs was <3 mm or <4 mm, respectively, the detection rate on panoramic radiographs was significantly decreased.

**Conclusion:**

CBCT is important for patients hoping to undergo implant-supported restorations of the maxilla because of the mucosal thickening in the maxillary sinus in such patients and their lower detection rates on panoramic radiographs.

## Introduction

Dental implant treatment is continually developing new methods to provide a better understanding of the biologic principles that direct the development of dynamic interference between the living tissue and the biomaterial. Furthermore, its techniques for missing teeth are common and can improve lower occlusion power more than other procedures such as dental bridges and dentures. However, dental implant surgery is relatively more invasive than other dental treatments such as endodontic procedures, and implant treatment failure has recently been reported [1-10]. In particular, in the maxilla, there might be some cases in which relatively difficult and invasive techniques are required, such as sinus floor elevation procedures and bone grafting. With these techniques, some complications have been reported [1-6], and a precise diagnosis is required before dental implant treatment planning.

In most dental offices in Japan, dental panoramic radiographs are commonly used as preoperative imaging evaluations to plan maxillary dental implants. Of course, this modality has been used clinically to evaluate the maxillary sinus of patients with maxillary dental implants. Dental panoramic radiographs are a more useful tool than dental radiographs for complete visualization of the maxillary sinus and evaluating the relationship between the level of the sinus floor and alveolar bone. However, they have a limitation for the three-dimensional (3D) visualization of anatomical structures because of their two-dimensional nature. In addition, soft tissues of the maxillary sinus cannot be effectively visualized on panoramic radiographs. There is as yet no precise understanding of the limitations of panoramic radiographs for the evaluation of anatomical variations and lesions of the maxillary sinus.

Computed tomography (CT) can visualize 3D structures and can provide precise information about complex anatomical structures. In particular, cone beam (CB) CT can precisely visualize teeth and surrounding anatomical structures with high resolution, despite the lower radiation dose levels than standard multi-detector row CT [11,12].

In the present study, the prevalence rates of anatomical variations and lesions in the maxillary sinus were evaluated in patients with maxillary dental implants using CBCT. The anatomical variations of the maxillary sinus that were evaluated included the presence of pneumatization and septa. Mucosal thickening, fluid retention, bone thickening, and sinus opacification related to the occurrence of maxillary sinusitis, discontinuity of the sinus related to perforations between the maxilla and sinus, and space occupying lesions (SOLs) such as retention cysts, polyps, and tumors were also evaluated. It is important to search for these because they are related to limitations in burying dental implants and are causes of worse inflammation after surgery. In particular, the lesions would be much worse if surgery failed. Next, the limitations of panoramic radiographs for the visualization of the maxillary sinus were evaluated based on the detection rates of anatomical variations and lesions on panoramic radiographs in comparison with the data using CBCT as a gold standard. At the same time, the weak points of panoramic radiographs for maxillary sinus evaluation were also examined by comparisons of the detection rates between the two modalities with respect to the locations and the height of septa, the extent of mucosal thickening, and the locations and sizes of SOLs.

## Materials and methods

**Figure 1:**
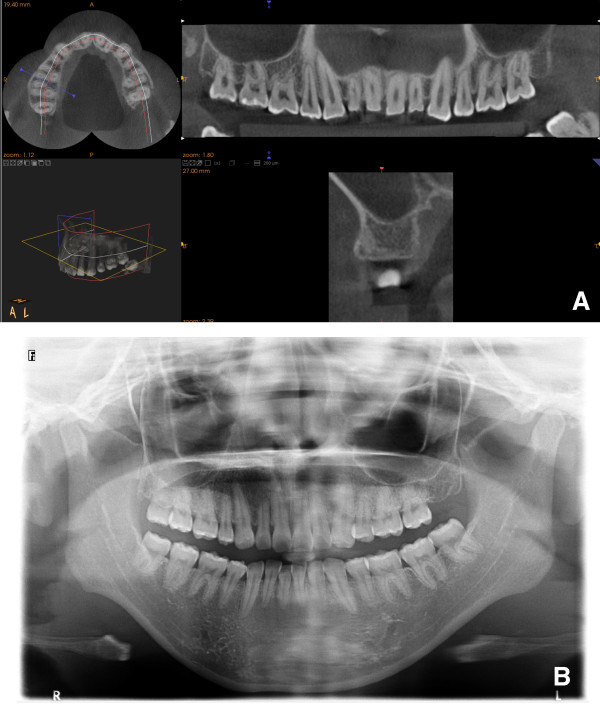
**Typical images of CBCT (A) and panoramic radiographs (B) in the present study.** The CBCT can visualize the area up to 35 mm above the patient’s occlusal plane and between the bilateral maxillary retromolar positions.

This study was based on 61 patients’ (107 sites) (13 males, 48 females; age range 20-87 years; mean age 56.6 ± 14.9 years) pairs of panoramic radiographs and CBCT scans, which were obtained at Yuugao Dental Office between 2011 and 2013 from two groups of patients; the patients of one group hoped to undergo implant-supported restorations of the maxilla (Implant group), while the remaining patients had other chief complaints (Non-implant group). The subjects were all patients in whom all areas of the maxilla including the maxillary sinuses were appropriately visualized on pairs of two images between 2011 and 2013. Of the 32 patients (55 sites) in the Implant group, 9 were males and 23 were females, ranging in age from 39 to 87 years (mean: 63.7 ± 10.5 years). Of the 29 patients (52 sites) in the Non-implant group, 4 were males and 25 were females, ranging in age from 20 to 77 years (mean: 48.6 ± 14.9 years). The reasons for CBCT without implant planning were pericoronitis of a maxillary third molar, potential for maxillary dentures, marginal and periapical periodontitis, pulpitis, supernumerary teeth, and displaced teeth. Approval of the present study was obtained from the institutional review board of Yuugao Dental Office (No. 12-0001).Panoramic radiographs were acquired using a panoramic radiographic machine (Trophypan plus, Carestream Health Co., Ltd., Rochester, NY, USA). Images were taken in the incisive occlusion position holding the head by an ear rod with the Frankfort plane parallel to the ground. CBCT was performed with a Trophypan plus (Carestream Health Co., Ltd.); 0.4-mm-thick sections were used to evaluate almost the same regions viewed on panoramic radiographs. Image analysis was performed on Trophy windows (Trophy Radiology Japan Co., Ltd, Tokyo, Japan), on a multiplanar reconstruction window in which the axial, coronal, and sagittal planes could be visualized at 0.4-mm intervals. The CBCT can visualize the area up to 35 mm higher from the patient’s occlusal plane and between the bilateral maxillary retromolar positions. Therefore, the areas that can be visualized on CBCT were evaluated in the present study. Typical CBCT imaging findings are shown in Figure 1.

The presence of the following anatomic variations and lesions was retrospectively examined on panoramic radiographs and CBCT: 1) pneumatization; 2) septa; 3) hypoplasia; 4) aplasia; 5) mucosal thickening; 6) SOLs (retention cysts, polyps, etc.); 7) discontinuity of the sinus floor; 8) fluid retention; 9) bone thickening; 10) antroliths; 11) exostoses; 12) sinus opacification; and 13) foreign bodies. In particular, bone thickening of the maxillary sinus on CBCT and panoramic radiographs was diagnosed based on the increase of thickness in the wall of the maxillary sinus. Exostoses were diagnosed based on the presence of high-density structures or radio-opacities on images. The distinction between a foreign body and an antrolith was made based on the extent of the density and opacity on images. For pneumatization, septa, and SOLs, changes in the detection rate on panoramic radiographs by location were examined to elucidate the weak points of visualization of the maxillary sinus on panoramic radiographs. At the same time, for septa, mucosal thickening, or SOLs, changes in the detection rate on panoramic radiographs according to increases in the height of septa, widths of mucosal thickening, or lengths of the major axis of SOLs were examined to elucidate the weak points of their visualization in the maxillary sinus on panoramic radiographs.

The imaging examinations were independently evaluated by two radiologists (K.S., and T.T.) who assessed the anatomic variations and lesions mentioned above. Of course, the locations and heights of septa, the width of mucosal thickening, and the major axes of SOLs were also evaluated. Disagreements among examiners were discussed and resolved by consensus. Each observer made two examinations with an interval of 1 week. The same order was always followed for the observations: first, the panoramic radiograph; second, the CBCT scan. When the evaluations were performed, the assessments from each observer were compared, and intra- and inter-observer agreements were calculated by the kappa test [13]. The kappa analysis was performed before the disagreements among examiners were discussed and resolved. Intra-observer agreement for detection using the kappa values was 0.90 for CBCT and 0.81 for panoramic radiographs. Inter-observer agreement for detection using the kappa values was 0.89 for CBCT and 0.80 for panoramic radiographs.

All statistical analyses were performed using SPSS version 11 statistical software (SPSS, Chicago, IL, USA). Categorical variables were compared by Fisher’s exact test. Relationships between categorical variables were assessed using Spearman’s correlation coefficient. Results were considered significant if p < 0.05.

## Results

### Prevalence of anatomic variations and lesions of the maxillary sinus in the two groups of patients

**Figure 2:**
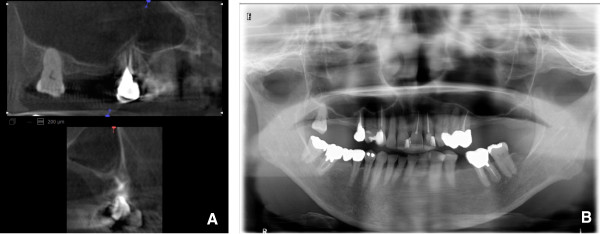
**Images of pneumatization in the maxillary sinus.** The CBCT **(A)** and panoramic radiograph **(B)** of a 68-year-old female undergoing preoperative planning for implantation of a left maxillary molar. The pneumatization is clearly visualized in the palatine area and anterior of the maxillary sinus on CBCT **(A)**, but not on panoramic radiographs **(B)**.

**Figure 3:**
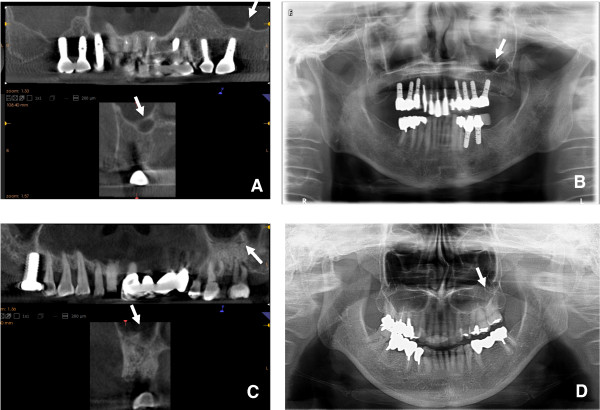
**Images of septa in the maxillary sinus.** The CBCT **(A)** and panoramic radiograph **(B)** of a 69-year-old female with septa >5 mm undergoing preoperative planning for implantation of a left maxillary molar. The CBCT **(C)** and panoramic radiograph **(D)** of a 73-year-old female with septa <5 mm undergoing preoperative planning for implantation of a left maxillary molar. The septa in the two patients are clearly visualized on CBCT **(A and C)**. However, the septa >5 mm are visualized on panoramic radiographs **(B)**, but those <5 mm are not **(D)**.

The detection rates of anatomic variations of the maxillary sinus in both groups of patients are shown in Table 1. The detection rate of pneumatization (Figure 2) in the maxillary sinus was relatively high, and that of septa (Figure 3) was also high. No significant differences in the presence of pneumatization (Fisher’s exact test; p = 0.518), septa (Fisher’s exact test; p = 0.383), hypoplasia (Fisher’s exact test; p = 0.262), and aplasia (Fisher’s exact test; p = 1.000) on CBCT were found between the two groups of patients.

**Table 1:** Detection rates of anatomic variations and lesions in the maxillary sinus by imaging modality in both groups of patient

**Anatomic variations and lesions of the maxillary sinus**	**Detection rates (Frequency)**					
	**With**		**Without**		**Total**	
	**Panoramic radiographs**	**CBCT**	**Panoramic radiographs**	**CBCT**	**Panoramic radiographs**	**CBCT**
Anatomic variations						
Pneumatization	38% (20/55)	42% (23/55)	40% (21/52)	40% (21/52)	38% (41/47)	41% (44/107)
Septa	33% (17/55)	51% (28/55)	29% (15/52)	46% (24/52)	30% (32/107)	49% (52/107)
Hypoplasia	4% (2/55)	4% (2/55)	0% (0/52)	0% (0/52)	2% (2/107)	2% (2/107)
Aplasia	0% (0/55)	0% (0/55)	0% (0/52)	0% (0/52)	0% (0/107)	0% (0/107)
Lesions						
Mucosal thickening	15% (8/55)	62% (32/55)	11% (6/52)	38% (20/52)	13% (14/107)	49% (52/107)
SOL	5% (3/55)	18% (10/55)	19% (10/52)	33% (17/52)	12% (13/107)	25% (27/107)
Discontinuity of the sinus floor	4% (2/55)	9% (5/55)	4% (2/52)	8% (4/52)	4% (4/107)	8% (9/107)
Fluid retention	0% (0/55)	0% (0/55)	0% (0/52)	0% (0/52)	0% (0/107)	0% (0/107)
Bone thickening	5% (3/55)	9% (5/55)	2% (1/52)	2% (1/52)	4% (4/107)	6% (6/107)
Antrolith	2% (1/55)	7% (4/55)	0% (0/52)	2% (1/52)	1% (1/107)	5% (5/107)
Exostosis	0% (0/55)	0% (0/55)	2% (1/52)	6% (3/52)	1% (1/107)	3% (3/107)
Sinus opacification	13% (7/55)	24% (13/55)	6% (3/52)	12% (6/52)	9% (10/107)	18% (19/107)
Foreign body	0% (0/55)	0% (0/55)	0% (0/52)	0% (0/52)	0% (0/107)	0% (0/107)

SOL: Space occupying lesion **p < 0.01.

CBCT: Come beam computed tomography *p < 0.05.

**Figure 4:**
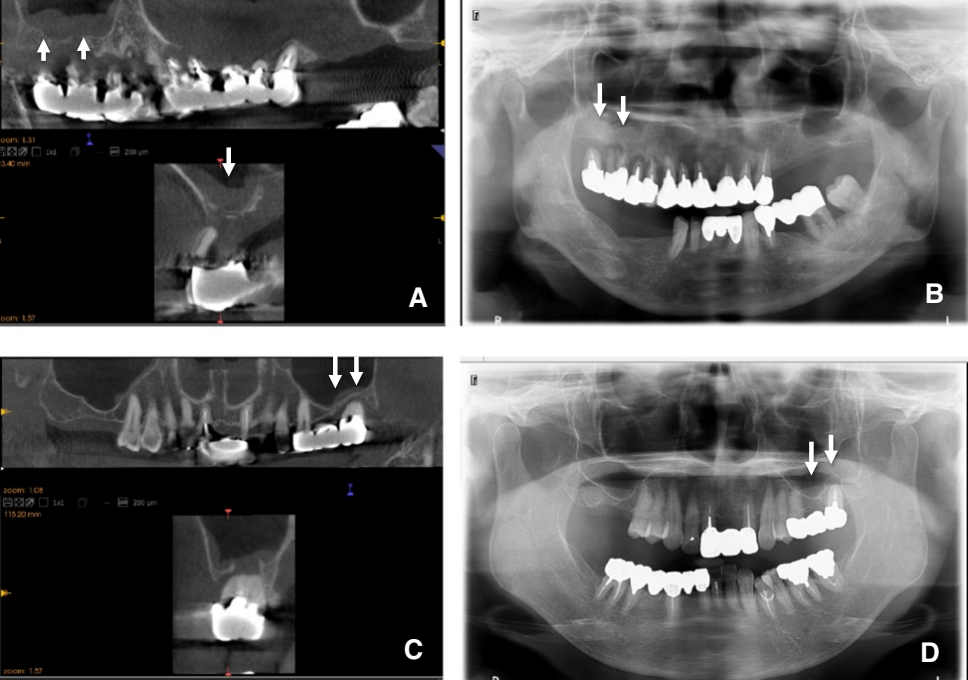
**Images of SOLs in the maxillary sinus.** The CBCT **(A)** and panoramic radiograph **(B)** of a 63-year-old female with an SOL with a major axis length >4 mm undergoing preoperative planning for implantation of a left maxillary molar. The CBCT **(C)** and panoramic radiograph **(D)** of a 70-year-old female with an SOL with a major axis length <4 mm undergoing preoperative planning for implantation of a left maxillary molar. The SOL is clearly visualized on CBCT **(A and C)**. However, the SOL with a major axis length >4 mm is visualized on panoramic radiographs **(B)**, but that < 4 mm is not **(D)**.

**Figure 5:**
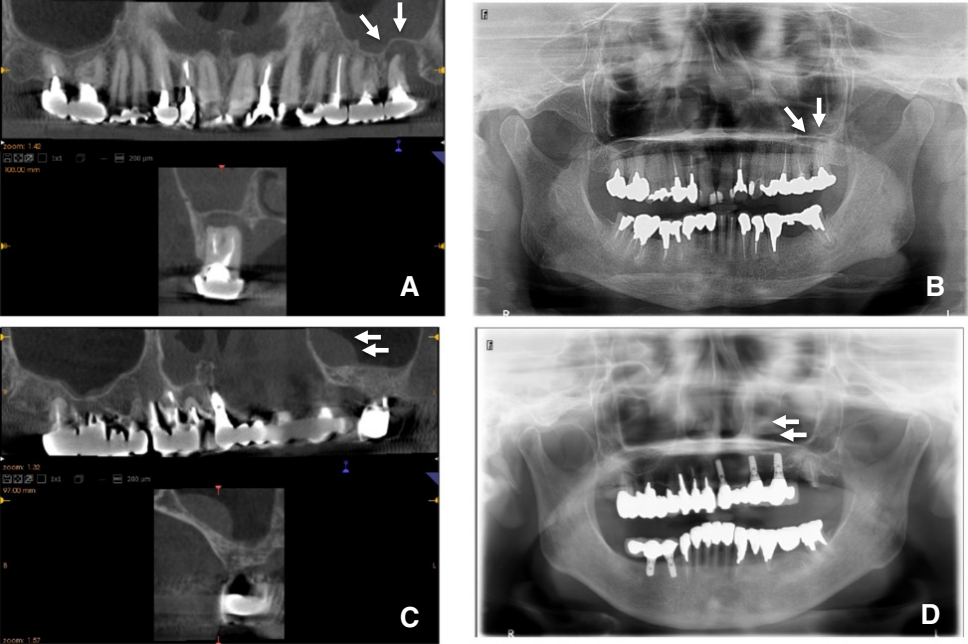
**Images of mucosal thickening in the maxillary sinus.** The CBCT **(A)** and panoramic radiograph **(B)** of a 58-year-old female with mucosal thickening >3 mm undergoing preoperative planning for implantation of a right maxillary molar. The CBCT **(C)** and panoramic radiograph **(D)** of a 66-year-old female with mucosal thickening <3 mm undergoing preoperative planning for implantation of a left maxillary molar. The mucosal thickening is clearly visualized on CBCT **(A and C)**. However, the lesion >3 mm is visualized on panoramic radiographs **(B)**, but that <3 mm is not **(D)**.

The detection rates of each lesion of the maxillary sinus in the two groups of patients are shown in Table 1. A significant difference was found in the presence of mucosal thickening (Fisher’s exact test; p = 0.032) (Figure 4) between the two groups. The detection rates were significantly higher in the Implant group. However, no significant differences were found in SOLs (Fisher’s exact test p = 0.066) (Figure 5), discontinuity of the sinus floor (Fisher’s exact test p = 0.536), fluid retention (Fisher’s exact test p = 1.000), bone thickening (Fisher’s exact test p = 0.140), antroliths (Fisher’s exact test p = 0.200), exostoses (Fisher’s exact test p = 0.111), sinus opacification (Fisher’s exact test p = 0.083), and foreign bodies (Fisher’s exact test; p = 1.000) between the two groups.

The imaging diagnoses of the SOLs and mucosal thickening on panoramic radiographs and CBCT were maxillary sinusitis, retention cysts, and radicular cysts (Table 2). About 60% of subjects had the various diseases mentioned above by imaging diagnosis (Table 2). No suspected malignancy was detected by imaging (Table 2).

**Table 2:** Imaging diagnosis of SOLs in the maxillary sinus by imaging modality in 61 patients

**Imaging diagnosis of SOLs in the maxillary sinus**	**Detection rate (Frequency)**	
	**Panoramic radiographs**	**CBCT**
Maxillary sinusitis	22% (24/107)	57% (61/107)
Retention cysts	6% (7/107)	17% (18/107)
Radicular cysts	6% (6/107)	8% (9/107)
Total	24% (26/107)	59% (63/107)

SOL: Space occupying lesion **p < 0.01.

CBCT: Cone beam computed tomography.

### The significance and limitations of panoramic radiographs in the visualization of anatomic variations and lesions in the maxillary sinus in the two patient groups

**Figure 6:**
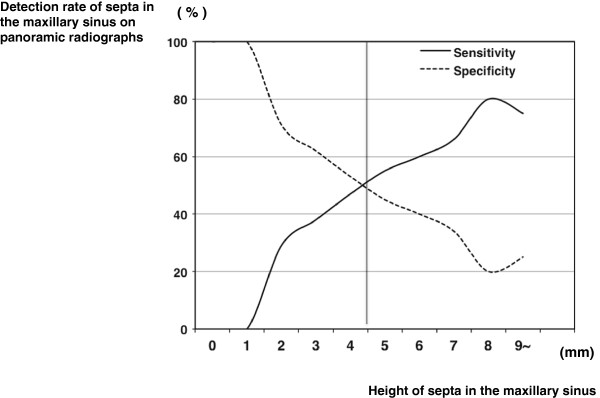
**The graph showing the relationship between the change in the detection rate on panoramic radiographs and maxillary sinus septal height.** The X-axis is the height of septa in the maxillary sinus. The Y-axis is the detection rate of septa in the maxillary sinus on panoramic radiographs. With decreasing height of the septa in the maxillary sinus, the detection rate on panoramic radiographs increases gradually. The threshold for less clear visualization of the septa is at a height of about 5 mm (arrow).

Panoramic radiographs showed significantly lower detection rates of almost all anatomic variations and lesions of the maxillary sinus including SOLs (Table 1). The detection rate of internally located pneumatizations was especially lower on panoramic radiographs (Fisher’s exact test; p = 0.030) (Table 3 and Figure 2). A similar tendency was observed for internally located septa (Fisher’s exact test; p < 0.001) (Table 3). The detection rate of anteriorly located SOLs was noticeably lower on panoramic radiographs (Fisher’s exact test; p = 0.001) (Table 3). A significant relationship was found between the change in the detection rate on panoramic radiographs and septal height in the maxillary sinus (Spearman’s correlation coefficient r = 0.542; p < 0.001) (Table 4). With decreasing height of septa in the maxillary sinus, the detection rate on panoramic radiographs decreased gradually (Table 4 and Figure 6). The threshold for clearer visualization of the septa by height was about 5 mm (Figures 3 and 6).

**Table 3:** Changes in the detection rates of pneumatization, septa, or SOLs according to the distributions in the maxillary sinus on panoramic radiographs in 61 patients

**Distributions**	**Detection rate of pneumatization (Frequency)**		**Detection rate of septa (Frequency)**		**Detection rate of SOLs (Frequency)**	
	**Panoramic radiographs**	**CBCT**	**Panoramic radiographs**	**CBCT**	**Panoramic radiographs**	**CBCT**
Alveolar (Foor)	33% (35/107)	36% (38/107)	21% (22/107)	37 (40/107)	18% (19/107)	21% (23/107)
Anterior	6% (6/107)	8% (9/107)	7% (8/107)	14% (15/107)	1% (1/107)	11% (12/107)
Tuber (Posterior)	10% (11/107)	13% (14/107)	3% (3/107)	7% (8/107)	1% (1/107)	5% (6/107)
Palatine (Internal)	0% (107)	5% (5/107)	0% (0/107)	14% (15/107)	0% (0/107)	1% (1/107)

SOL: Space occupying lesion **p < 0.01.

CBCT: Cone beam computed tomography *p < 0.05.

**Table 4:** Changes in the detection rates of septa according to the height of septa in the maxillary sinus on panoramic radiographs in 61 patients

**Height of septa in maxillary sinus on CBCT**	**Detection rate of panoramic radiographs (Frequency)**
0 < <=1 mm	0% (0/1)
1 < <=2 mm	29% (2/7)
2 < <=3 mm	30% (7/23)
3 < <=4 mm	38% (5/13)
4 < <=5 mm	47% (8/17)
5 < <=6 mm	55% (5/9)
6 < <=7 mm	50% (2/4)
7 < <=8 mm	66% (2/3)
8 < <=9 mm	-
9 < <=10 mm	100% (1/1)
10 < mm	66% (2/3)

CBCT: Cone beam computed tomography.

**Figure 7:**
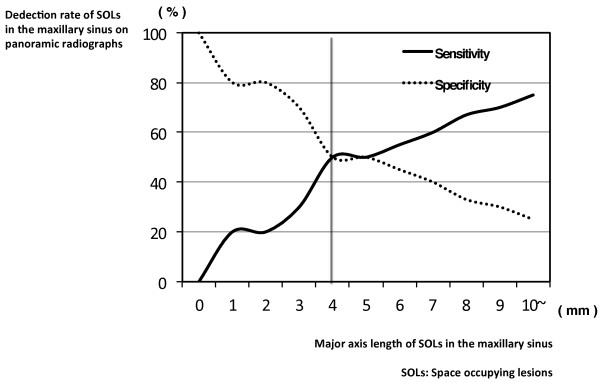
**Graph showing the relationship between the change in the detection rate on panoramic radiographs and the major axis length of SOLs in the maxillary sinus.** The X-axis is the major axis length of SOLs in the maxillary sinus. The Y-axis is the detection rate of SOLs in the maxillary sinus on panoramic radiographs. With decreases of the major axis length of SOLs in the maxillary sinus, the detection rate of SOLs on panoramic radiographs decreases gradually. If the major axis length of SOLs is <4 mm, the detection rate of SOLs on panoramic radiographs decreases significantly (arrow).

At the same time, a significant relationship was found between the change in the detection rate on panoramic radiographs and the length of the major axis of SOLs (Spearman’s correlation coefficient r = 0.575; p < 0.001) (Table 5). With a decreasing major axis of SOLs in the maxillary sinus, the detection rate on panoramic radiographs decreased gradually (Table 5 and Figure 7). In particular, if the length of the major axis of SOLs was <4 mm, the detection rate on panoramic radiographs was significantly lower (Figures 5 and 7).

**Table 5:** Changes in the detection rates of SOLs according to the extent of SOLs in the maxillary sinus on panoramic radiographs in 61 patients

**Major axis of SOLs in maxillary sinus on CBCT**	**Detection rate of Panoramic radiographs (Frequency)**
0 < <=1 mm	20% (1/5)
1 < <=2 mm	0% (0/1)
2 < <=3 mm	25% (1/4)
3 < <=4 mm	50% (1/2)
4 < <=5 mm	33% (1/3)
5 < <=6 mm	50% (3/6)
6 < <=7 mm	60% (3/5)
7 < <=8 mm	67% (4/6)
8 < <=9 mm	50% (1/2)
9 < <=10 mm	25% (1/4)
10 < mm	83% (5/6)

SOL: Space occupying lesion.

CBCT: Cone beam computed tomography.

**Figure 8:**
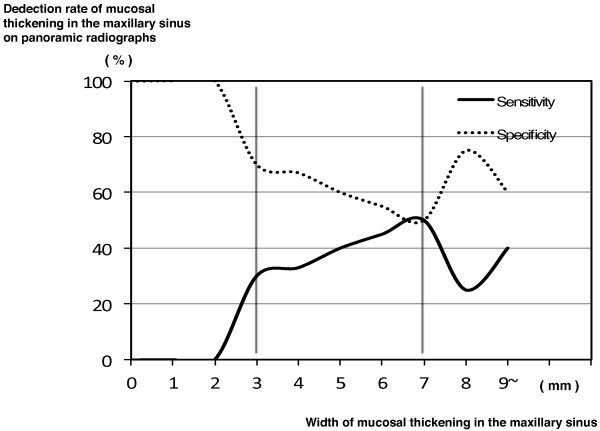
**Graph showing the relationship between the change in the detection rate on panoramic radiographs and the widths of mucosal thickening in the maxillary sinus.** The X-axis is the width of mucosal thickening in the maxillary sinus. The Y-axis is the detection rate of mucosal thickening in the maxillary sinus on panoramic radiographs. With decreasing widths of mucosal thickening in the maxillary sinus, the detection rate of mucosal thickening on panoramic radiographs decreases gradually. If the width of mucosal thickening is <3 mm, the detection rate of mucosal thickening on panoramic radiographs decreases significantly (arrow). If the width of mucosal thickening ranges from 7 to 10 mm, the detection rate of mucosal thickening in the maxillary sinus on panoramic radiographs decreases. With increasing widths of mucosal thickening over 10 mm in the maxillary sinus, the detection rate on panoramic radiographs increases gradually.

**Figure 9:**
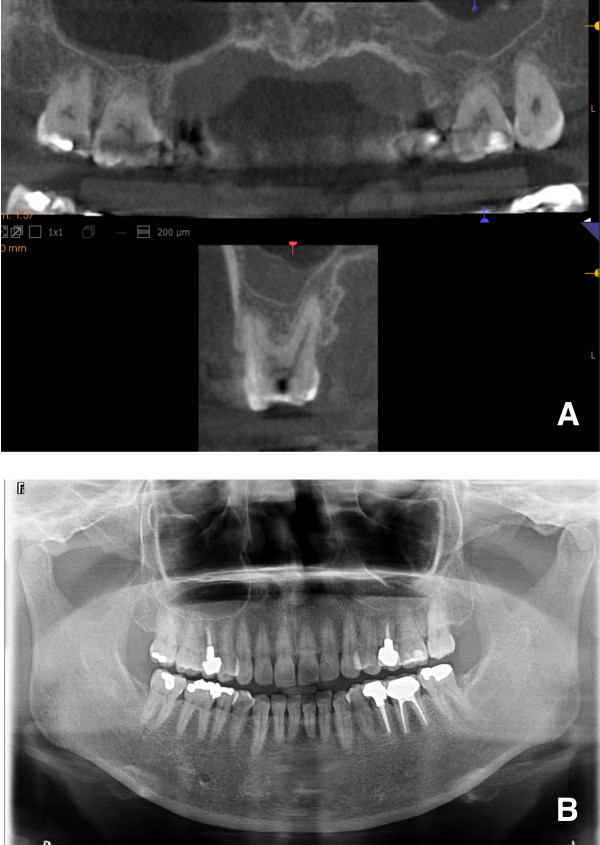
**Images of mucosal thickening in the maxillary sinus.** The CBCT **(A)** and panoramic radiograph **(B)** of a 45-year-old female with mucosal thickening of about 7 mm undergoing preoperative planning for implantation of a left maxillary molar. The mucosal thickening is clearly visualized on CBCT **(A)**, but not on the panoramic radiograph **(B)**.

A significant relationship was found between the change in the detection rate on panoramic radiographs and the width of mucosal thickening in the maxillary sinus (Spearman’s correlation coefficient r = 0.371; p < 0.001) (Table 6). With decreasing widths of mucosal thickening from 0 to 7 mm in the maxillary sinus, the detection rate on panoramic radiographs decreased gradually (Table 6 and Figure 8). In particular, if the width of mucosal thickening was <3 mm, the detection rate on panoramic radiographs was significantly lower (Figures 4 and 8). If the widths of mucosal thickening ranged from 7 to 10 mm, the detection rate of mucosal thickening in the maxillary sinus on panoramic radiographs decreased (Table 6 and Figure 8). The reason for this phenomenon might be superimposition between the hard and soft palates and the line of mucosal thickening (Figure 9). With increased width of mucosal thickening over 10 mm in the maxillary sinus, the detection rate on panoramic radiographs increased gradually (Table 6 and Figure 8).

**Table 6:** Changes in the detection rates of mucosal thickening according to the extent of mucosal thickening in the maxillary sinus on panoramic radiographs in 61 patients

**Widths of mucosal thickening in maxillary sinus on CBCT**	**Detection rate of Panoramic radiographs (Frequency)**
0 < <=1 mm	0% (0/1)
1 < <=2 mm	0% (0/8)
2 < <=3 mm	30% (3/10)
3 < <=4 mm	33% (2/6)
4 < <=5 mm	40% (2/5)
5 < <=6 mm	20% (1/5)
6 < <=7 mm	50% (1/2)
7 < <=8 mm	25% (1/4)
8 < <=9 mm	-
9 < <=10 mm	0% (0/4)
10 < mm	67% (4/6)

CBCT: Cone beam computed tomography.

## Discussion

In some cases undergoing treatment planning for implant-supported restorations in the maxilla, the bone quantity may be deficient for implantation because of absorption of alveolar bone and the presence of the maxillary sinus. Relatively invasive techniques, such as floor elevation procedures and maxillary sinus lifts, are required for these cases, and some complications have been reported [1-10]. Therefore, diagnosis of lesions in the maxillary sinus should be precisely and fully done to assess bone quality, bone quantity, and anatomical complexity before treatment planning. It is very important to pay attention to imaging of the maxillary sinus. In fact, symptoms frequently do not appear at the outset of some lesions in the maxillary sinus. Therefore, the diagnosis is often made accidentally when images of the area are obtained for other purposes. As one of the important results of the present study, the patients hoping to undergo implant-supported restorations of the maxilla had a two times greater prevalence of maxillary sinusitis than patients with a chief complaint other than implant planning. The possible explanation for this finding may be that most patients whose maxillas need dental implant treatment have missing maxillary teeth because of inflammatory lesions such as pulpitis and periapical and/or periodontal inflammation(s) [14,15]. Thus, maxillary sinusitis may occur more commonly in such patients. Of course, there were some causes of maxillary sinusitis other than odontogenic inflammation, such as rhinogenous and allergic inflammations. Therefore, maxillary sinusitis was detected in the patients of both groups. However, there were no significant differences in the detection rates of anatomic variations and other diseases except for maxillary sinusitis between the two groups. Furthermore, the present rates of various kinds of anatomic variations and abnormalities were similar to those in previous reports [16-21]. Therefore, the present patients appeared representative.

The presence of pneumatization of the maxillary sinus as an anatomic variation is related to limitations in burying dental implants due to lack of bone in the maxilla. Similarly, the presence of septa in the maxillary sinus limits dental implant-related surgeries such as maxillary sinus lift. In the present study, the detection rates of pneumatization and septa in the maxillary sinus were about 40% and 50%, respectively. At the same time, panoramic radiographs clearly had limitations. In particular, the detection rates of internally located pneumatizations and septa and of anteriorly located SOLs were almost 0% or 1% and very low on panoramic radiographs. These results were similar to those in previous reports [16,17,20]. The respective walls of the maxillary sinus were not equal to the respective walls of the maxillary sinus on panoramic radiographs, except for the floor [22]. The so-called anterior wall on panoramic radiographs shows the transitional area from anterior to internal, and the so-called posterior wall shows that area from posterior to internal [22]. Therefore, a possible explanation was that the respective walls of the maxillary sinus except the sinus floor could not be expressed as a tangential line on panoramic radiographs.

In addition, with decreasing height of the septa in the maxillary sinus, the detection rate on panoramic radiographs decreased gradually. At the same time, the threshold for visualization of the septa occurred at a height of about 5 mm. The present data are very valuable because there are many septa with a low height that cannot be visualized on panoramic radiographs. The threshold for poorer visualization of mucosal thickening of the maxillary sinus and the presence of SOLs in the maxillary sinus could be determined based on the size or height, as for septa in the maxillary sinus. For example, if mucosal thickening of the maxillary sinus floor were <3 mm, visualization on panoramic radiographs was unlikely. If mucosal thickening of the maxillary sinus were detectable, the condition should be judged as chronic maxillary sinusitis based on diagnostic criteria [23]. Therefore, one could diagnose it as chronic maxillary sinusitis with the detection of mucosal thickening by panoramic radiographs. If the length of the major axis of SOLs in the maxillary sinus were <4 mm, they would not be easy to visualize on panoramic radiographs. Moreover, if the widths of mucosal thickening ranged from 7 to 10 mm, the detection rate of mucosal thickening in the maxillary sinus on panoramic radiographs decreased greatly. The phenomenon of mucosal thickening did not occur in SOLs. The possible explanation was that mucosal thickening of 7-10 mm in the maxillary sinus would tend to result in superimposition of the line of mucosal thickening on the panoramic radiograph with the hard and soft palates. Panoramic radiographs should be a relatively useful tool for the detection of maxillary sinus lesions, because many lesions occur from the sinus floor in the maxillary sinus [24]. In particular, the modality was very useful for the visualization of the relationship between teeth and the floor of the maxillary sinus. However, the beginning of lesions could not be visualized by this modality, and panoramic radiographs have a limitation in the visualization of lesions in the maxillary sinus, as in previous reports [17,18,25,26]. Thus, CT should be added to dental panoramic radiographs for evaluation of the maxillary sinus. In the present study, fortunately, there were no patients with malignancies suspected on CBCT in the maxillary sinus, which may have serious consequences for patient survival. CT and CBCT should be used clinically for pre-operative evaluations during planning for implant-supported restorations in the maxilla. The government should support the spread and maintenance of the low cost of CBCT. However, it has been reported that the majority of dentists ordered panoramic radiographs alone (63.8%) or in association with other radiographic methods (28.9%) for dental implant diagnosis [22]. Based on our empirical knowledge, many dentists in dental offices in Japan use panoramic and dental radiographs, but not CT, to plan for implant-supported restorations in the maxilla. Based on this and previous reports, this is a very dangerous strategy due to the occurrence of complications during maxillary dental implantation [17,18,20]. In our opinion, CT should be added to dental panoramic radiographs for evaluation of the maxillary sinus.

The limitation of this study is that the sample size was not large, and the subjects were all patients in a private dental office. Dental implants are expensive, and the present results should be interpreted as reflecting phenomena in relatively healthy, active, and rich populations. In addition, our CBCT had a limitation for visualizing areas in the upper maxillary sinus. Therefore, it was not possible to precisely evaluate the detection rate of anatomical variations and some kinds of diseases in the upper maxillary sinus. At the same time, data about the anterior and posterior walls only in the lower maxillary sinuses were collected in the present study.

## Conclusion

The purpose of the present study was to elucidate the significance of CBCT for patients hoping to undergo implant-supported restorations of the maxilla. Therefore, two studies were planned. One was to evaluate the prevalence of anatomic variations and lesions in the maxillary sinus of patients hoping to undergo implant-supported restorations of the maxilla on CBCT in a private dental office in Japan. The other was to elucidate the limitations of panoramic radiographs in the detection of anatomic variations and lesions of the maxillary sinus. It was found that sinusitis was significantly more prevalent in the Implant group than in the Non-implant group. Panoramic radiographs have limitations in the visualization of the maxillary sinus. The limitations depended on the distributions and the sizes of anatomical variations and lesions. In fact, if the width of mucosal thickening or the length of the major axis of SOLs was <3 mm or <4 mm, respectively, the detection rate on panoramic radiographs was significantly decreased.

In conclusion, based on the results of the present study, it is our view that CBCT should be required for treatment planning for implant-supported restorations of the maxilla because of the higher prevalence of anatomical variations and mucosal thickening in such patients and their lower detection rates on panoramic radiographs.

### Clinical significance

CBCT should be required for treatment planning for implant-supported restorations in the maxilla because of the higher prevalence of anatomical variations and mucosal thickening in such patients and their lower detection rates on panoramic radiographs.

### Ethical approval

Approval of the present study was obtained from the institutional review board of Yuugao Dental Office (No. 12-0001).

## Competing interests

There are no potential conflicts of interest to disclose.

## Authors’ contributions

KS, TT, and YM: Conceptualized and designed the study, performed research, analyzed the data, and drafted the initial manuscript. KS and TT: Performed research and collected data. TT, KS, NW, SM, MO, and SN: Drafted the initial manuscript, critically reviewed the manuscript, and approved the final manuscript as submitted. All authors read and approved the final manuscript.
